# Factor V has an anticoagulant cofactor activity that targets the early phase of coagulation

**DOI:** 10.1074/jbc.M116.769570

**Published:** 2017-04-18

**Authors:** Salvatore Santamaria, Natalia Reglińska-Matveyev, Magdalena Gierula, Rodney M. Camire, James T. B. Crawley, David A. Lane, Josefin Ahnström

**Affiliations:** From the ‡ Centre for Haematology, Faculty of Medicine, Imperial College London, London W12 0NN, United Kingdom,; the § Division of Hematology, Department of Pediatrics, Perelman School of Medicine, University of Pennsylvania, Philadelphia, Pennsylvania 19104, and; the ¶ Center for Cell and Molecular Therapeutics, Children's Hospital of Philadelphia, Philadelphia, Pennsylvania 19104

**Keywords:** coagulation factor, enzyme inhibitor, inhibition mechanism, phospholipid, protein-protein interaction, FV, TFPI, anticoagulant, protein S, tissue factor pathway inhibitor

## Abstract

Tissue factor pathway inhibitor (TFPI), the main inhibitor of initiation of coagulation, exerts an important anticoagulant role through the factor Xa (FXa)-dependent inhibition of tissue factor/factor VIIa. Protein S is a TFPI cofactor, enhancing the efficiency of FXa inhibition. TFPI can also inhibit prothrombinase assembly by directly interacting with coagulation factor V (FV), which has been activated by FXa. Because full-length TFPI associates with FV in plasma, we hypothesized that FV may influence TFPI inhibitory function. Using pure component FXa inhibition assays, we found that although FV alone did not influence TFPI-mediated FXa inhibition, it further enhanced TFPI in the presence of protein S, resulting in an ∼8-fold reduction in *K_i_* compared with TFPI alone. A FV variant (R709Q/R1018Q/R1545Q, FV^ΔIIa^) that cannot be cleaved/activated by thrombin or FXa also enhanced TFPI-mediated inhibition of FXa ∼12-fold in the presence of protein S. In contrast, neither activated FV nor recombinant B-domain-deleted FV could enhance TFPI-mediated inhibition of FXa in the presence of protein S, suggesting a functional contribution of the B domain. Using TFPI and protein S variants, we show further that the enhancement of TFPI-mediated FXa inhibition by protein S and FV depends on a direct protein S/TFPI interaction and that the TFPI C-terminal tail is not essential for this enhancement. In FXa-catalyzed prothrombin activation assays, both FV and FV^ΔIIa^ (but not activated FV) enhanced TFPI function in the presence of protein S. These results demonstrate a new anticoagulant (cofactor) function of FV that targets the early phase of coagulation before prothrombinase assembly.

## Introduction

TFPI inhibits the initiation of coagulation through the factor Xa (FXa)-dependent^2^ inhibition of the tissue factor-activated factor VII complex (1, 2). TFPI is a Kunitz-type inhibitor comprising an acidic N terminus, three Kunitz domains, and a basic C terminus (3). The first Kunitz domain (K1) binds to and inhibits activated factor VII (3), and K2 binds to and inhibits FXa (4). Because the TFPI concentration necessary for efficient inhibition of FXa (5) is higher than the free full-length TFPI concentration in plasma (0.25–0.5 nm) (6, 7), TFPI is on its own a poor inhibitor of FXa. Protein S functions as a cofactor for TFPI, efficiently enhancing TFPI inhibition of FXa (8, 9) and the inhibition of FXa activation by TF-VIIa (10). The protein S enhancement of TFPI is dependent on a direct interaction involving Glu-226 (9) and Arg-199 (11) within the TFPI K3 domain and laminin G-like domain 1 of the protein S sex hormone-binding globulin (SHBG)-like region (12, 13).

TFPI has been shown to bind to FV (14, 15), FVa (15, 16), and two alternatively spliced FV variants, FV-short (16, 17) and FV Amsterdam (18). FV is a 330-kDa multidomain (A1-A2-B-A3-C1-C2) procofactor that has little or no intrinsic procoagulant function until its conversion to FVa (19). This occurs through limited proteolysis by thrombin or FXa at Arg-709, Arg-1018, and Arg-1545, resulting in the removal of the B domain (19). FVa and FXa are part of the prothrombinase complex on phospholipid surfaces that is essential for the rapid generation of thrombin (19). FV also has an anticoagulant function by acting as a synergistic cofactor together with protein S in the activated protein C-mediated inactivation of FVIIIa (20).

Within the FV B-domain, there are an acidic and a basic region that interact with each other, maintaining FV in an inactive procofactor conformation (16, 21–23). A basic sequence within the TFPI C terminus is highly homologous to a basic sequence within the FV B-domain, enabling a high-affinity interaction of TFPI when the acidic region is exposed, such as in FV-short and partially activated FV (16, 17). Early studies showed that FVa enhanced FXa inhibition by TFPI (5) and that FV enhanced FXa inhibition by TFPI at high TFPI concentrations (24). However, in a more recent study, FVa protected, rather than enhanced, FXa from inhibition by TFPI (25). Moreover, TFPI can efficiently bind partially activated forms of FV (retaining the acidic region) and thereby block prothrombinase assembly (16) and conversion to FVa (26). The same finding was observed for FV810 (16), a recombinant B-domain-deleted FV similar to FV-short (27). Both FVa (28–31) and FV (29) can directly interact with protein S, suggesting that FV might influence both TFPI and protein S function.

Although several studies have investigated the role of different forms of FV in the TFPI pathway, little is known regarding how these FV derivatives influence the cofactor function of protein S in the TFPI-mediated inhibition of FXa. We demonstrate herein that FV (but not FVa or FV810) enhances the TFPI cofactor function of protein S.

## Results

### Enhancement of TFPI-mediated inhibition of FXa by protein S and FV

**Figure 1.**
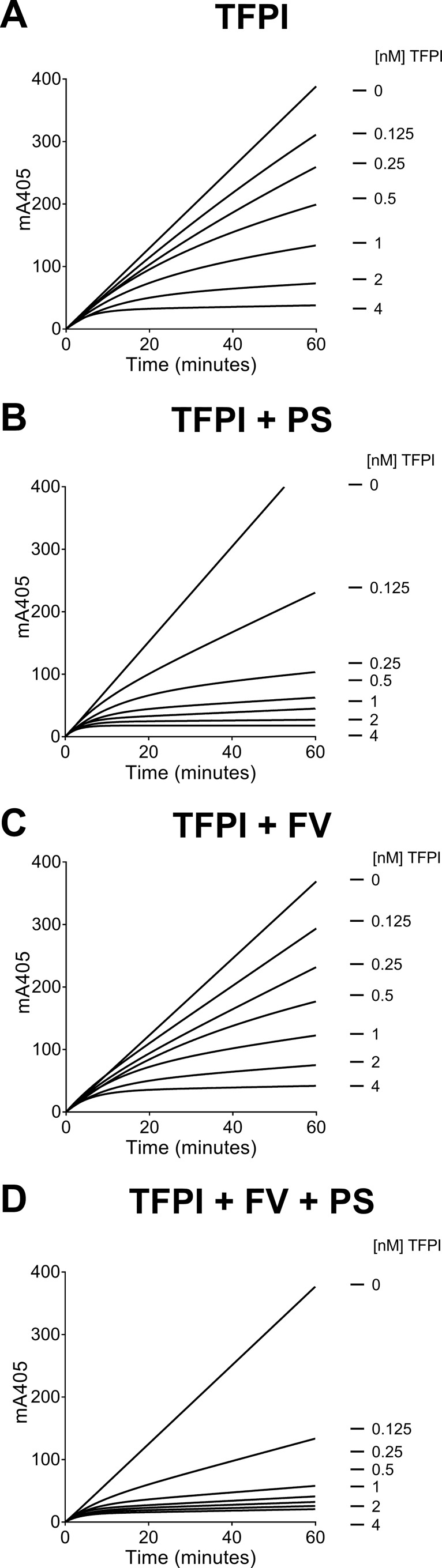
**Enhancement of TFPI-mediated inhibition of FXa by protein S and FV.** *A–D*, TFPI (0–4 nm) alone (*A*) or in the presence of 100 nm protein S (*B*), 30 nm FV (*C*), or 30 nm FV and 100 nm protein S (*D*) was incubated with 200 μm substrate S-2765, 25 μm of phospholipids vesicles (DOPC/DOPS/DOPE, 60:20:20), and 5 mm CaCl_2_. FXa (0.3 nm) was used to initiate the reaction, and FXa activity was measured through cleavage of the chromogenic substrate at 405 nm over 60 min. The results from a representative experiment (*n* = 5) are shown. *PS*, protein S.

The influence of protein S and/or FV in the inhibition of FXa by TFPI was analyzed by inhibition assays measuring cleavage of a chromogenic substrate by FXa in real time. As previously reported (8, 9), TFPI dose-dependently inhibited FXa (Fig. 1*A*). This inhibition was further enhanced by physiological concentration (100 nm) of its cofactor, protein S (Fig. 1*B*). In contrast with previous studies using plasma-purified FV (ppFV) (24, 25), our recombinant FV (30 nm) had no effect upon TFPI inhibition of FXa in the absence of protein S (Fig. 1, compare *A* and *C*). However, when FV was added in combination with protein S, it further enhanced FXa inhibition (Fig. 1, compare *B* and *D*, and Table 1).

**Table 1:** *K_i_* and K_i_* for the inhibition of FXa by TFPI in the presence and absence of protein S (100 nm) and FV (30 nm) The results are given in nm and expressed as means ± S.E. PS, protein S.

	*K_i_*		K_i_*	
	TFPI	TFPI + PS	TFPI	TFPI + PS
	*nm*		*nm*	
No FV	2.71 ± 0.32	0.70 ± 0.07*^a^*	0.034 ± 0.01	0.016 ± 0.003
FV	3.87 ± 052	0.36 ± 0.09*^a^*^,^*^b^*	0.060 ± 0.02	0.008 ± 0.002
FV^ΔIIa^	1.7 ± 0.42	0.23 ± 0.08*^a^*^,^*^c^*	0.037 ± 0.01	0.013 ± 0.004
FVa	3.96 ± 0.22*^d^*	1.87 ± 0.72*^c^*	0.16 ± 0.064*^d^*	0.019 ± 0.006
FV810	7.16 ± 2.6*^d^*	2.23 ± 0.16*^e^*	0.021 ± 0.003	0.040 ± 0.027

*^a^ p* < 0.0001 compared to TFPI, according to Mann-Whitney tests (*n* = 4–19).

*^b^ p* < 0.05 compared to TFPI + PS, according to Mann-Whitney tests (*n* = 4–19).

*^c^ p* < 0.005 compared to TFPI + PS, according to Mann-Whitney tests (*n* = 4–19).

*^d^ p* < 0.05 compared to TFPI, according to Mann-Whitney tests (*n* = 4–19).

*^e^ p* < 0.0005 compared to TFPI + PS, according to Mann-Whitney tests (*n* = 4–19).

The inhibition of FXa by TFPI occurs as a two-step process, where TFPI K2 first forms an encounter complex with FXa (FXa-TFPI), followed by a slow isomerization into a final tight complex (FXa-TFPI*) (5).

(Reaction. 1) FXa+TFPI⇆FXa/TFPI⇆FXa/TFPI*

The dissociation constant for the encounter complex is described as *K_i_*, whereas K_i_* represents an overall dissociation constant. As previously described (8, 9), the protein S cofactor effect is mainly manifest through the enhancement of the formation of the FXa-TFPI encounter complex (*K_i_*). Protein S reduces the *K_i_* from 2.71 to 0.70 nm (Table 1). This enhancement brings the *K_i_* for FXa inhibition toward the plasma concentration of full-length TFPI (0.25–0.5 nm) (6, 7). Addition of FV further augmented FXa inhibition by TFPI (∼8-fold compared with TFPI alone), resulting in a *K_i_* of 0.36 nm (Table 1), which, importantly, is much closer to the plasma concentration of full-length TFPI. Protein S also reduced the K_i_* from 0.034 to 0.016 nm and the combination of FV and protein S gave an overall 5-fold enhancement (from 0.034 to 0.008 nm), compared with TFPI alone. As shown in Fig. 1*D*, the enhancement by FV was most apparent at physiological concentrations (<0.5 nm) of full-length TFPI.

**Figure 2.**
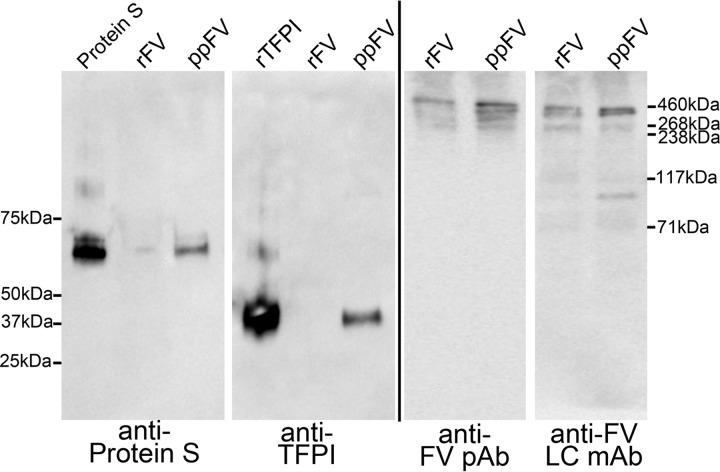
**Comparison between recombinant and plasma-purified FV.** Recombinantly produced in house FV (*rFV*) or ppFV were loaded on a 4–12% gel (4.95 μg/lane), blotted, and probed either with a polyclonal anti-human protein S antibody or a mixture of monoclonal antibodies directed against the K1, K2, and K3 domains and the C terminus of TFPI. As standard, 2 ng of recombinant TFPI and 69 ng of PS were used. For comparison, immunoblots of recombinant FV and ppFV (16.5 ng/lane) were probed either with a polyclonal antibody against FV (*anti-FV pAb*) or a monoclonal antibody directed against the light chain of FV (*anti-FV LC mAb*). Note that the monoclonal antibody has higher affinity for cleaved FV rather than full-length FV, as stated in the manufacturer's instructions.

Because of the aforementioned discrepancy between our results and previous observations (24, 25), we compared the enhancement of TFPI inhibition by our recombinant FV with commercial ppFV. Similar to previous reports (24, 25) and in contrast to recombinant FV, we did observe protein S-independent enhancement of TFPI inhibition by ppFV. However, the magnitude of this enhancement was variable between different batches of ppFV (data not shown). Because cleavage of FV or small amounts of contaminating TFPI and protein S in the FV batches has the potential to significantly alter the results and conclusions of our study, we assessed the quality of the FV using Western blotting. Although the recombinant FV was essentially uncleaved, the ppFV showed variable amounts of cleavage, but more importantly, several of the tested ppFV batches contained appreciable amounts of protein S and minor amounts of TFPI, which were not present in our recombinant preparation. Representative Western blots of one batch of ppFV compared with our recombinant FV are shown in Fig. 2. Because of these results, we considered the recombinant FV more suitable for this investigation.

**Figure 3.**
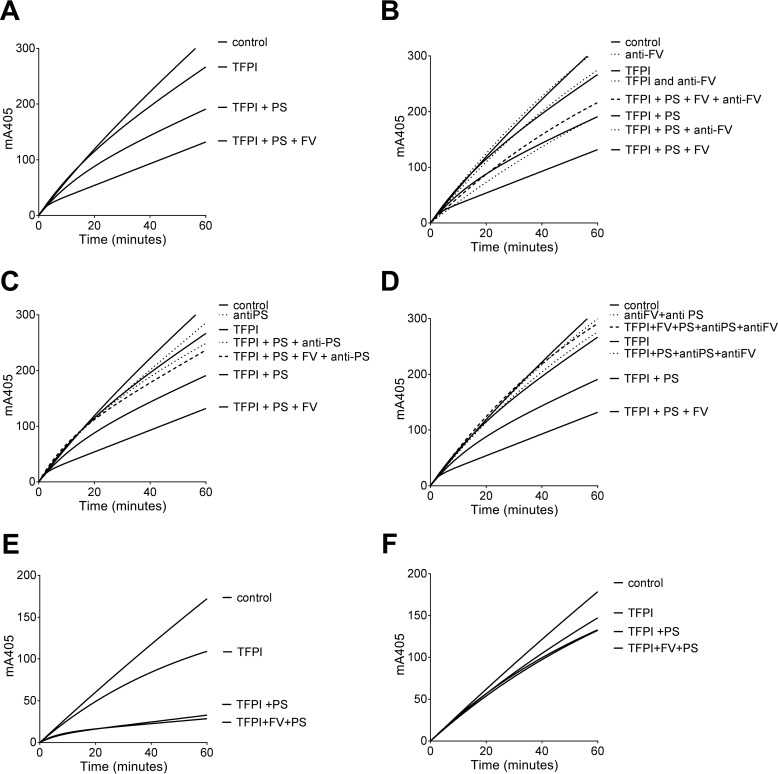
**Enhancement of TFPI-mediated inhibition of FXa by protein S and FV is specific and phospholipid-dependent.** *A–D*, FXa activity (0.3 nm) was followed in real time through cleavage of S-2765 (200 μm) at 405 nm in the presence of 25 μm phospholipids and the presence or absence of 0.25 nm TFPI, 100 nm protein S, and 30 nm FV (*A*). The same experiment was performed in the presence of anti-FV (300 nm, *B*), anti-protein S (1000 nm, *C*), and anti-FV (300 nm) + anti-protein S (1000 nm, *D*) antibodies. *E* and *F*, FXa activity (0.3 nm) was followed in real time through cleavage of S-2765 (200 μm) at 405 nm in the presence or absence of 1 nm TFPI, 100 nm protein S and 30 nm FV, and in the presence (*E*) or absence (*F*) of 25 μm phospholipids (DOPC/DOPS/DOPE, 60:20:20). *PS*, protein S. *Control* indicates reactions performed in the absence of TFPI, protein S, FV, and antibodies. Representative experiments are shown (*n* = 3).

To verify the specificity of FV cofactor function in FXa inhibition by TFPI and protein S, anti-protein S and anti-FV antibodies (10-fold excess) were preincubated with recombinant FV and protein S for 10 min before addition of TFPI and FXa (Fig. 3, *A–D*). Neither monoclonal antibodies against the FV light chain nor polyclonal antibodies against protein S influenced FXa activity when added alone or in the presence of TFPI. However, anti-FV antibodies abolished the TFPI enhancement by FV in the presence of protein S (Fig. 3*B*) but did not influence protein S cofactor function. Anti-protein S antibodies prevented the enhancement by both protein S and FV (Fig. 3, *C* and *D*), demonstrating that the cooperative effect exerted by FV and protein S was specific and highly dependent upon protein S.

To examine the phospholipid dependence of FV cofactor function, higher (1 nm) concentrations of TFPI were used because of its decreased inhibitory function in the absence of phospholipids (5, 8). Under these conditions, FXa activity was completely inhibited after addition of TFPI, protein S, and FV in the presence of phospholipids (Fig. 3*E*). However, no enhancement of TFPI was detected for either protein S or FV in the absence of phospholipids (Fig. 3*F*). These results suggest that, like the TFPI enhancement of protein S alone (8), the enhancement of TFPI in the inhibition of FXa by protein S and FV is phospholipid-dependent.

### FV activation abolishes its protein S-dependent TFPI cofactor function

Because FXa can activate FV into FVa (19) and early reports (5, 24) showed that FVa can enhance TFPI inhibition of FXa, we investigated the effects of FVa on TFPI-mediated inhibition of FXa (Table 1). FVa significantly increased the *K_i_* for TFPI inhibition of FXa from 2.71 to 3.96 nm, demonstrating that FVa partially protects FXa from TFPI inhibition. FVa also increased the K_i_* for TFPI inhibition in the absence of protein S (from 0.034 to 0.16 nm). In the presence of protein S and FVa, there was a modest, non-significant reduction in *K_i_* (from 2.71 to 1.87 nm). We then tested recombinantly expressed B domain-deleted FV (FV810) (27). As for FVa, a similar increase in the *K_i_* for FXa inhibition was observed by FV810 alone (from 2.71 to 7.16 nm). In contrast, FV810 in the absence of protein S had no effect upon K_i_*. Together, these results suggest that the augmentation of protein S enhancement of the TFPI cofactor effect of FV and protein S requires non-activated FV. To confirm this result, we substituted all three thrombin-FXa cleavage sites in FV (residues Arg-709, Arg-1018, or Arg-1545) for glutamine to generate FV^ΔIIa^. This variant was then tested in the FXa inhibition assay and found to increase the enhancement of TFPI by protein S 12-fold, compared with TFPI alone (from 2.71 to 0.23 nm) (Table 1), similar to FV.

### Structural requirements for FV cofactor function

To examine the mechanisms underlying FV cofactor function, both TFPI and protein S variants were used in FXa inhibition assays. TFPI ΔCT (aa 1–249), lacking the basic region of the C-terminal tail, and TFPI E226Q, a variant with appreciably diminished ability to bind and be enhanced by protein S (9), were studied (Table 2). Similar to previously described TFPI variants (TFPI 1–252 and TFPI 1–247) (32, 33), TFPI ΔCT showed decreased inhibitory potency against FXa compared with full-length TFPI (*K_i_*, 5.69 *versus* 2.71 nm). Although the *K_i_* for FXa inhibition by TFPI ΔCT was not significantly influenced by FV alone (7.41 nm) or protein S alone (4.57 nm), in the presence of both FV and protein S, it was enhanced by ∼3.5-fold (*K_i_* = 1.61 nm). This suggests that the basic TFPI C-terminal tail is not strictly required for the protein S-dependent TFPI cofactor function by FV. However, it is required for optimal interactions between TFPI, protein S, FV, and FXa. TFPI E226Q inhibition of FXa was not enhanced by protein S or FV, either separately or in combination, suggesting that the interaction between TFPI and protein S is required for FV cofactor function (Table 2).

**Table 2:** *K_i_* and K_i_* for the inhibition of FXa by TFPI ΔCT and TFPI E226Q in the presence and absence of PS (100 nm) and FV (30 nm) The results are given in nm and expressed as means ± S.E. PS, protein S.

	TFPI ΔCT				TFPI E226Q			
	*K_i_*		K_i_*		*K_i_*		K_i_*	
	TFPI	TFPI + PS	TFPI	TFPI + PS	TFPI	TFPI + PS	TFPI	TFPI + PS
	*nm*				*nm*			
No FV	5.69 ± 0.96	4.57 ± 0.68	0.050 ± 0.012	0.047 ± 0.016	2.23 ± 0.47	2.61 ± 0.78	0.021 ± 0.007	0.028 ± 0.010
FV	7.41 ± 1.18	1.61 ± 0.35*^a^*^,^*^b^*	0.31 ± 0.21	0.028 ± 0.009	4.38 ± 0.78	2.07 ± 0.55	0.025 ± 0.008	0.022 ± 0.011

*^a^ p* < 0.005 compared to TFPI + PS, according to Mann-Whitney tests (*n* = 6–7).

*^b^ p* < 0.005 compared to TFPI + PS, according to Mann-Whitney tests (*n* = 6–7).

The TFPI/protein S interaction is dependent on the SHBG-like region of protein S (12). The same region in protein S has also been suggested to be involved in the interaction with FV (30). Using protein S chimera III (12), a variant in which the entire protein S SHBG-like region (Val-243–Ser-635) was substituted by the corresponding region in growth arrest specific protein 6 (34), we studied the role of the protein S SHBG region on the inhibition of FXa by TFPI in the presence and absence of FV. We have reported that this variant is unable to directly interact with TFPI (12). As previously shown, protein S chimera III did not enhance FXa inhibition by TFPI (*K_i_* = 2.87 nm; Table 3) when compared with the absence of protein S (*K_i_* = 2.16 nm). Moreover, FV was unable to significantly enhance TFPI in the presence of this protein S chimera III (*K_i_* 1.49 nm, *p* > 0.05). This corroborates the contention that the TFPI K3 interaction with protein S SHBG region is required for the FV-mediated augmentation of protein S enhancement of TFPI.

**Table 3:** *K_i_* and K_i_* for the inhibition of FXa by TFPI in the presence and absence of protein S chimera III (100 nm) and FV (30 nm) The results are given in nm and expressed as means ± S.E. (*n* = 4). PS, protein S.

	PS chimera III			
	*K_i_*		K_i_*	
	TFPI	TFPI + PS	TFPI	TFPI + PS
	*nm*		*nm*	
No FV	2.16 ± 0.85	2.87 ± 1.78	0.065 ± 0.048	0.047 ± 0.016
FV	2.03 ± 0.66	1.49 ± 0.54	0.042 ± 0.015	0.072 ± 0.028

### Enhancement of TFPI-mediated inhibition of FXa-catalyzed prothrombin activation by protein S and FV

A physiological consequence of FXa inhibition by TFPI is reduced prothrombin activation. Whereas TFPI efficiently inhibits prothrombin activation by FXa in the absence of FVa, it exhibits minimal inhibition of prothrombin activation when FVa is present (16, 24, 25). We therefore investigated whether the protein S-dependent enhancement we observed by FV is also present when prothrombin is the substrate.

**Figure 4.**
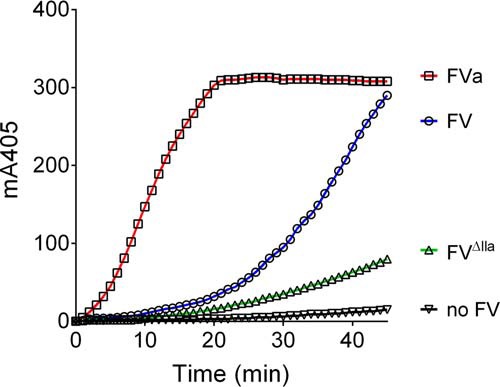
**Effect of FV, FV^ΔIIa^, and FVa on thrombin generation by FXa.** Different FV forms (200 pm) were incubated with 1 μm prothrombin, 117 μm substrate S-2238, 25 μm of phospholipids vesicles (DOPC/DOPS/DOPE, 60:20:20), and 5 mm CaCl_2_ before addition of FXa (0.6 pm). Thrombin activity was measured through cleavage of the chromogenic substrate at 405 nm over time.

Prothrombin activation was initiated by adding FXa to prothrombin, phospholipid vesicles, a thrombin-specific chromogenic substrate, and either FV, FV^ΔIIa^, or FVa. We first compared efficiency of prothrombin activation by FXa (0.6 pm) in the presence or absence of these FV forms (each at 200 pm) (Fig. 4). As expected, FVa enhanced the activation of prothrombin by FXa effectively, as shown by the increase in prothrombin activation compared with FXa alone. After ∼20 min the absorbance reached a plateau, reflecting depletion of the chromogenic substrate for thrombin. FV was able to enhance prothrombin activation after a lag phase (∼20 min) during which small amounts of activated FV were generated by FXa or thrombin. In contrast, because of its resistance to be cleaved and activated by FXa, FV^ΔIIa^ showed minor enhancement of prothrombin activation compared with FXa alone. We estimated that between 5 and 15 min, the rate of prothrombin activation was 0.3% in the absence of FV and 7 and 3.5% in the presence of FV and FV^ΔIIa^, respectively, compared with the presence of FVa. The prothrombin activation appeared to follow a biphasic pattern in the presence of wild-type FV. This agrees with FV being activated into FVa during the assay, where the rate of prothrombin to thrombin conversion at 30–40 min is similar to that of FVa.

**Figure 5.**
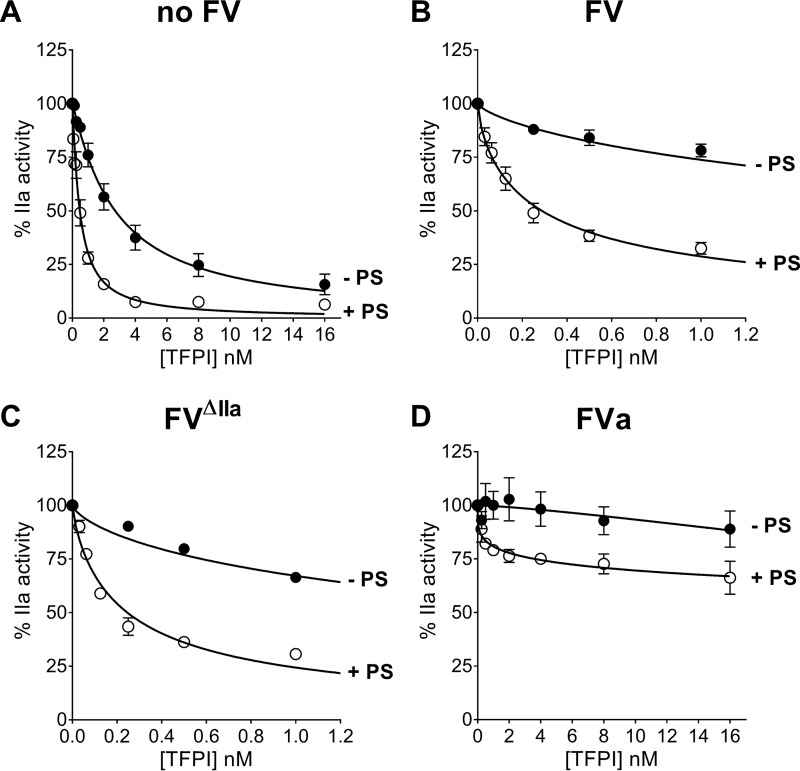
**Enhancement of TFPI-mediated inhibition of thrombin generation by FXa in the presence or absence of FV and protein S.** TFPI (0–16 nm) in the absence (●) or presence (○) of 100 nm protein S in the absence of FV (*A*) and in the presence of 200 pm FV (*B*), FV^ΔIIa^ (*C*), or FVa (*D*) was added to a prewarmed (10 min, 37 °C) solution containing 1 μm prothrombin, 117 μm substrate S-2238, 25 μm of phospholipids vesicles (DOPC/DOPS/DOPE, 60:20:20), and 5 mm CaCl_2_. FXa (60 pm (*A*), 0.6 pm (*B* and *C*), or 0.06 pm (*D*)) was used to initiate the reaction, and thrombin activity was measured through cleavage of the chromogenic substrate at 405 nm over time. Thrombin formation was expressed as a percentage of the rate obtained in the absence of TFPI. The results are expressed as means ± S.E. (*n* = 4–7). Note the *x* axis scale change in *B* and *C* compared with *A* and *D* to better show the enhancement by FV and PS at lower TFPI concentrations. For each curve, only the linear range of curve was used for measuring thrombin activity as stated in the text and under “Experimental procedures.”

Because of the varying efficiency of prothrombin activation, we used different concentrations of FXa to analyze TFPI inhibition in the presence FVa (FXa concentration, 0.06 pm) or FV or FV^ΔIIa^ (FXa concentration, 0.6 pm) each at 200 pm concentration or in the absence of FV (FXa concentration, 60 pm). Therefore, in these experiments, similarly to those using FXa chromogenic substrate, FV was present in large excess over FXa. The slopes of the linear portions of these progress curves, representing rates of thrombin formation, were expressed as percentages of the rate obtained in the absence of TFPI and plotted as function of the TFPI concentration (Fig. 5). For wild-type FV, only the linear portion of the initial phase, before substantial conversion to FVa occurred, was chosen for analysis (see “Experimental procedures” for details). TFPI (0–16 nm) dose-dependently inhibited FXa-catalyzed prothrombin activation in the absence of FV (Fig. 5*A*), as previously described (25). Similar to the direct FXa inhibition assay, TFPI inhibitory function was further enhanced by 100 nm protein S. The IC_50_ values^3^ for TFPI inhibition (2.94 nm), as well as the 6-fold enhancement by protein S (0.51 nm; Table 4), were very close to the *K_i_* values we measured for inhibition of FXa peptidolytic activity (Table 1).

**Table 4:** **IC_50_ values for the inhibition of FXa by TFPI in the presence and absence of protein S and FV, assessed using prothrombinase assays** The results are given in nm and expressed as means ± S.E. NI, not inhibited; NA, not applicable; PS, protein S.

	IC_50_		
	TFPI	TFPI + PS	Fold enhancement by protein S
	*nm*		
No FV*^a^*	2.94 ± 0.68	0.51 ± 0.1*^b^*	5.8
FV*^a^*	4.12 ± 0.50	0.25 ± 0.04*^c^*	16.5*^d^*
FV^ΔII^*^a^*^,^*^e^*	2.79 ± 0.19	0.25 ± 0.02*^c^*	11.2*^d^*
FVa*^f^*	NI	NI	NA

*^a^* 60 pm FXa.

*^b^ p* < 0.05 compared to TFPI according to Mann-Whitney tests.

*^c^ p* < 0.005 compared to TFPI according to Mann-Whitney tests.

*^d^ p* < 0.05 compared to TFPI + PS according to Mann-Whitney tests (*n* = 4–7).

*^e^* 200 pm FV and 0.6 pm FXa.

*^f^* 200 pm FVa and 0.06 pm FXa.

The enhancement of TFPI-mediated inhibition of prothrombin activation by protein S was even more pronounced in the presence of either FV (Fig. 5*B*) or FV^ΔIIa^ (Fig. 5*C*), 16.5- and 11.2-fold, respectively (Table 4). As previously shown, FXa-catalyzed prothrombin activation was very poorly inhibited by TFPI in the presence of FVa alone (25). The inhibition was also very modest in combination with protein S (34% inhibition; Fig. 5*D*), suggesting that FXa is protected against inhibition by TFPI in the presence of prothrombin and FVa, independent of whether protein S is present or not.

## Discussion

TFPI is an important inhibitor of coagulation, regulating the initiation phase of thrombin generation, as well as reducing the rate of thrombin generation during the propagation phase (35). Importantly, the decrease in thrombin generation by TFPI has also been shown to be associated with a delay in FV activation (26, 35). In this study, we used FXa inhibition assays to evaluate the influence of protein S and FV on TFPI-mediated inhibition of FXa peptidolytic activity. Using purified recombinant TFPI, protein S, and FV, within the range of their respective plasma concentrations, we found that protein S alone enhanced TFPI inhibitory function in the presence of phospholipids with a *K_i_* value similar to that previously reported by our lab (9). Somewhat surprising, unlike two previous reports using ppFV (24, 25), we did not detect any significant enhancement of FXa inhibition by TFPI when recombinant FV was added in the absence of protein S (Fig. 1*C*). Our data suggest that the different results are due to the sources of FV used. We identified contaminating TFPI and protein S in several ppFV batches, potentially influencing the results when using ppFV (Fig. 2).

The major enhancement of TFPI by protein S and FV was observed for *K_i_* rather than K_i_*. This reduction in the *K_i_* suggests that together, protein S and FV function by enhancing the formation of the TFPI-FXa encounter complex, most likely by approximating enzyme and inhibitor on negatively charged phospholipid surfaces. Once the initial TFPI-FXa complex is formed, the slow isomerization (as measured by the K_i_*) into the tight complex appears to be less affected by protein S and FV. Although we found no significant enhancement of TFPI by FV alone, we detected an 8-fold (for wild type FV) or 12-fold (for FV^ΔIIa^) enhancement of FXa inhibition by TFPI in the presence of both FV and protein S. FV consistently further augmented the inhibitory function of TFPI and protein S between 2-fold (wild-type FV) and 3-fold (FV^ΔIIa^). Is this relatively modest enhancement likely to contribute to TFPI inhibition *in vivo*? In the absence of cofactors, the inhibition constant for FXa inhibition by TFPI (Table 1) is well above the physiological full-length TFPI concentration (0.25–0.5 nm) (6). In our hands, in the presence of protein S, the *K_i_* is still above or at most within the range of these values (0.7 nm) (Table 1 and Ref. 9). However, the addition of FV brings the *K_i_* value down to or below the physiological full-length TFPI concentration.

To further evaluate the molecular mechanisms underlying the cooperative enhancement of TFPI-mediated FXa inhibition by protein S and FV, we used previously characterized TFPI and protein S variants (12). We demonstrated that if the interaction between the protein S SHBG-like region and TFPI K3 is abolished (by using protein S chimera III, anti-protein S antibodies, or TFPI E226Q), FV can no longer augment protein S-mediated enhancement of TFPI inhibition of FXa (Tables 2 and 3). These findings reveal that the ability of FV to function in this system is absolutely dependent upon the TFPI-protein S interaction. There could be two possible mechanisms underlying FV enhancement of TFPI-protein S. FV could augment TFPI cofactor function by protein S through a scaffolding effect in which FV directly interacts with both TFPI and protein S. Alternatively FV may influence the affinity of the TFPI-protein S complex through interaction with one of these proteins. Although augmentation of FXa inhibition by protein S and FV was highly dependent upon phospholipids (Fig. 3*F*), it is unclear whether FV binding to phospholipid surfaces is necessary or whether FV mediates its enhancing effects through interaction(s) with protein S and/or TFPI.

Interestingly, the protein S SHBG-like region has been suggested to contain an interaction site for FV (30). In addition, protein S chimera III cannot support FV-enhanced inactivation of FVIIIa by activated protein C. This could therefore potentially suggest that similar protein S SHBG-like region-dependent interactions with FV occur in the two anticoagulant pathways (36).

Using different forms of FV (FV, FV^ΔIIa^, FV810, and FVa), we found that whereas full-length FV exhibits protein S-dependent TFPI cofactor function, this anticoagulant function of FV is lost upon activation by thrombin to FVa, which results in the removal of the entire B domain (Table 1). Again, this observation reflects the activated protein C cofactor function of FV in FVIIIa degradation, which is also protein S-dependent (20). The activated protein C cofactor function of FV requires the C-terminal portion of its B-domain, which is removed upon conversion to FVa (20, 37, 38). We found that FVa (and FV810) not only lost its cofactor function for TFPI inhibition of FXa but that it also protected FXa in the prothrombinase complex from TFPI-mediated inhibition (Table 1). This result is consistent with a recent observation (25) and with early studies reporting protection of FXa from antithrombin inactivation in the presence of FVa (39, 40).

Because only full-length FV (as opposed to FVa or FV810) exhibits a cofactor effect, this may suggest a functional contribution of the FV B domain. The basic region within the TFPI C-terminal tail has been shown to be involved in the interaction with FXa-activated FV and FV-short via interaction with an acidic region in the C-terminal end of the B domain (17, 26). It has also been suggested to be involved in the interaction with FV and FVa (15). However, because the TFPI basic region within the C-terminal tail is dispensable for the cooperative cofactor function by protein S and FV (Table 2), it is likely that any cofactor interaction between TFPI and FV is mechanistically distinct from the binding between TFPI and FV-short or FXa-activated FV. This is perhaps not surprising given that the acidic region within full-length FV is most likely not exposed or available for binding TFPI (21).

The enhancement of TFPI-mediated inhibition of FXa by FV and protein S was also investigated in the context of prothrombin activation. In agreement with recently published results (11, 25), TFPI successfully inhibited prothrombin activation by FXa in the absence of FVa, whereas FVa protected FXa from TFPI inhibition (Table 4 and Fig. 5). Inhibition of FXa-mediated prothrombin activation was enhanced by protein S in the absence of FV-FVa, as well as in the presence of FV and FV^ΔIIa^. However, the protein S enhancement was more efficient in the presence of both full-length forms of FV, confirming the results observed in the FXa inhibition assays. It is worth noting that we did not observe direct inhibition of prothrombinase by protein S when FXa was added to initiate the reaction. However, we found that protein S inhibits prothrombinase when it was preincubated with FXa, FVa, and phospholipids, and the reaction was initiated by the addition of prothrombin (data not shown). These results are in agreement with previously published reports (28, 42–44).

Because of the protecting effects of FVa on FXa activity, it is most likely that the TFPI cofactor functions of protein S and FV are relevant during the initiation phase of coagulation, prior to when significant FV activation takes place. TFPI is known to prolong the initiation phase of coagulation, as well as delay FV activation. Therefore, even a modest (<10-fold) enhancement of TFPI has the potential to have a major effect on coagulation. Once coagulation reaches the propagation phase, in which prothrombin is quantitatively activated (45), increasing amounts of FVa are produced, which assemble with FXa into prothrombinase complexes that are resistant from the action of TFPI. FV activation serves to convert FV from an anticoagulant to a procoagulant cofactor and ensures that FV-FVa does not exhibit its anticoagulant and procoagulant functions simultaneously. Defining the role that the FV B-domain plays in this cofactor function will provide a more refined model of the molecular basis for this mechanism. Moreover, this will potentially enable studies to be rationally designed to specifically target the anticoagulant functions of FV and thus assess its importance in a more physiological setting.

In summary, we have identified a protein S-dependent TFPI cofactor function of protein S and FV in the inhibition of FXa peptidolytic activity, as well as in the prothrombin activation by FXa. This cofactor function is dependent on phospholipids, as well as an optimal TFPI/protein S interaction, and is likely to be important physiologically to enable the low, subnanomolar concentrations of full-length TFPI to exert its important anticoagulant effects.

## Experimental procedures

### Expression and purification of TFPI, protein S, and FV

TFPI and protein S variants were expressed in mammalian cells, purified, and quantified as previously described (9, 12, 46). Purified recombinant TFPI was estimated by ELISA (9) to be 88–100% full-length. TFPI ΔCT (aa 1–249) was a kind gift from Dr. Tsatumo Hamuro (Chemo-Sero-Therapeutic Research Institute, Kaketsuken, Japan). FV^ΔIIa^ (R709Q/R1018Q/R1545Q) was generated by site-directed mutagenesis, using the wild-type FV vector as a template (27). The different FV variant vectors (pED) were stably transfected into BHK cells and expressed as described previously (27).

Concentrations of different FV forms were measured using *A*_280_ (*E*_280_ 0.1%: 9.6 for wild-type FV and FV^ΔIIa^ and 1.54 for FV810) as previously described (27). The concentrations of FV and FV810 were confirmed by prothrombinase assays (41), using thrombin-activated ppFV (Hematologic Technologies Inc., HTI) for reference. Concentration of FV^ΔIIa^ was confirmed by semiquantitative Western blotting using polyclonal sheep anti-FV (HTI) and known concentrations of ppFV as standards.

For experiments involving FVa, FV was activated with human α thrombin (Enzyme Research Laboratories), and the reaction was stopped with hirudin (Sigma), as previously described (45). Control experiments were run to ensure that the activation reagents did not influence the assays (data not shown).

### SDS-PAGE and Western blotting

Proteins were run under non-reducing conditions on a 4–12% Mini-Protean TGX gradient gels (Bio-Rad) and electroblotted using the Trans-Blot® Turbo^TM^ transfer system (Bio-Rad) at 25 V for 30 min. Blots were acquired using a Chemidoc^TM^ imaging system (Bio-Rad) and analyzed using the Image Lab^TM^ software. Monoclonal mouse antibodies against K1, K2, and K3 and the C terminus of TFPI were purchased from Mast Group Ltd., polyclonal rabbit anti-protein S was from Dako, and monoclonal mouse anti-FV light chain antibody 5112 and polyclonal sheep anti-FV were from Hematologic Technologies Inc.

### Phospholipid vesicle preparation

The phospholipids (Avanti Polar Lipids) 1,2-dioleoyl-*sn*-glycero-3-phosphocholine (DOPC), 1,2-dioleoyl-*sn*-glycero-3-phosphoserine (DOPS), and 1,2-dioleoyl-*sn*-glycero-3-phosphoethanolamine (DOPE) were mixed in a molar ratio of 60:20:20 and extruded as previously described (46).

### Inhibition of FXa peptidolytic activity by TFPI

FXa (0.3 nm; HTI) activity was monitored by following the cleavage of S-2765 (200 μm; Chromogenix) for 60 min at 25 °C using the absorbance at 405 nm, in the presence of 0–16 nm TFPI, 0–100 nm protein S, 0–30 nm FV, 25 μm phospholipids, and 5 mm CaCl_2,_ as described previously (9, 11). All data sets were repeated using three or four different batches of each recombinantly produced protein. The FXa concentration was determined based on its specific activity through cleavage of S-2765. As controls, experiments were also run in the presence and absence of polyclonal anti-human protein S (1000 nm; Dako) or anti-FV light chain 5101 (300 nm; HTI) antibodies.

The inhibition of FXa by TFPI occurs as a two-step process (5), according to reaction 1, where *K_i_* and K_i_* represent the dissociation constants for the formation of the encounter complex (FXa-TFPI) and the overall interaction, respectively.

The initial (*v*_0_) and steady-state velocities (*v*_s_) of S-2765 cleavage were determined, and the inhibitory constants for the FXa/TFPI interaction, *K_i_* and K_i_*, respectively (Equation 1), were calculated from a plot of *V*/*v*_0_ *versus* the concentration of TFPI, in which *V* is the rate of product formation by FXa in the absence of TFPI. The *x* intercept of this line is as follows,

(Eq. 1) $$x=-K_{i}/\left(1+\left[S\right]/K_{m}\right)$$

where [S] is the concentration of chromogenic substrate S-2765, and *K_m_* is the Michaelis-Menten constant (8).

The *K_m_* values for the hydrolysis of S-2765 by FXa were determined in the presence of 25 μm phospholipids, 5 mm CaCl_2_, and 0–5 mm chromogenic substrate and in the presence or absence of FV, FVa, FV810, and protein S. The *K_m_* values (± S.E.) were similar to previously described (5): 45 ± 3 μm in the absence of FV, 46 ± 3 μm with mock-activated FV, 98 ± 5 μm with FVa, and 89 ± 13 μm with FV810 (*n* = 3). Protein S and full-length FV did not affect the *K_m_* (data not shown).

### Inhibition of FXa-catalyzed prothrombin activation by TFPI

Prothrombin activation by FXa was analyzed in a similar manner to a previous report (25). Thrombin-specific chromogenic substrate S-2238 (117 μm; Chromogenix), 25 μm DOPC/DOPS/DOPE (60:20:20), 5 mm CaCl_2_, and 1 μm prothrombin (Enzyme Research Laboratories) were preincubated for 7 min at 37 °C in TBS containing 0.5% BSA. Subsequently, prewarmed TFPI (0–16 nm) and sequentially prewarmed FXa were added, and the reaction was followed at 405 nm for 60 min at 37 °C. FXa concentrations were 60 pm (in the absence of FV), 0.6 pm FXa (in the presence of 200 pm FV and FV^ΔIIa^), or 0.06 pm (in the presence of 200 pm FVa). For these experiments, commercially available FVa (not containing thrombin or hirudin, HTI) was used. When added, 100 nm protein S were present in the reaction mixture. In the presence of FV, a biphasic progress curve was observed, which was characterized by a lag phase (10–20 min), followed by an exponential phase once FV was activated to FVa (21–50 min; Fig. 4). In this case, to specifically assess the enhancement in the presence of FV and not FVa, only the first linear phase was chosen for analysis. From the linear portion of the progress curves (5–20 min in the absence of FV, 10–20 min for wild-type FV and FV^ΔIIa^, and 5–15 min for FVa), thrombin generation by unit of time was determined and converted to percentage of thrombin activity compared with the reaction in the absence of TFPI (100%). IC_50_ values (*i.e.* TFPI concentrations giving 50% residual activity) were determined using the formula,

(Eq. 2) $$v_{\text{i}}/v=1/\left(1+\left[\text{I}\right]/{\text{IC}}_{50}\right)$$

where *v*_i_ is the initial velocity of substrate cleavage in the presence of the inhibitor at concentration [I], and *v* is the initial velocity in the absence of the inhibitor.

### Statistical analysis

The results were analyzed using Prism software (GraphPad Software, La Jolla, CA), version 6.0. All of the data were analyzed by Mann-Whitney test. *p* values <0.05 were considered as significant.

## Author contributions

S. S., J. T. B. C., D. A. L., and J. A. designed the research, analyzed the results, and wrote the paper. S. S., N. R.-M., and M. G. performed the experiments. R. M. C. contributed essential reagents. All authors reviewed the results, contributed to the data interpretation, and approved the final version of the manuscript.

## Footnotes

3 IC_50_ is defined as the TFPI concentration giving 50% residual FXa/prothrombinase activity.

## Footnotes

2 The abbreviations used are:

FXa: activated factor X
FV: factor V
FVa: activated FV
FVIIIa: activated factor VIII
K1: K2, and K3, Kunitz domains 1, 2, and 3
ppFV: plasma purified FV
SHBG: sex hormone-binding globulin
TFPI: tissue factor pathway inhibitor
DOPC: 1,2-dioleoyl-*sn*-glycero-3-phosphocholine
DOPS: 1,2-dioleoyl-*sn*-glycero-3-phosphoserine
DOPE: 1,2-dioleoyl-*sn*-glycero-3-phosphoethanolamine.
